# Microbiological Sealing Analysis of a Tapered Connection and External Hexagon System

**DOI:** 10.1155/2017/3849085

**Published:** 2017-02-28

**Authors:** Gardel Nepomuceno Costa, Elizabeth Ferreira Martinez, Aluísio Martins de Oliveira Ruellas, Daiane Cristina Peruzzo, Júlio Cesar Joly, Marcelo Henrique Napimoga

**Affiliations:** São Leopoldo Mandic Institute and Research Center, Campinas, SP, Brazil

## Abstract

Considering the variety of implant connection systems available in the market and the contrasting literature regarding tapered connection systems in terms of bacterial leakage, the aim of this in vitro study was to compare the effectiveness of the bacterial seal at the implant/abutment interface between an external hexagon and a tapered connection system. Twelve sets of indexed tapered connection components and twelve sets of external hexagon connection components were used for microbiological analysis. In addition, for each model, an implant with its respective prosthetic abutment was used as a negative control and another as a positive control of microbial contamination. Failure of the abutment/implant interface seal was observed via turbidity or presence of deposits in the culture. Descriptive analysis of the data and relative frequency (percentage) as well as Fisher's exact test were used at a significance level of 5%. Two of ten (20%) external hexagon specimens showed contamination against 0/10 (0%) tapered connection implants. In conclusion, both implant/abutment connections were able to prevent bacterial leakage in vitro.

## 1. Introduction

One of the limiting factors in the success of implant therapy is the inherent presence of an oral microbiota that can lead to persistent peri-implant infection. As most implant systems are composed of two pieces; peri-implantitis can develop despite osseointegration [1, 2].

The microgap between implant and prosthetic abutment allows leakage and exchange of fluids as well as bacteria originating from the tissue fluid and saliva between the inner part of the implant and the oral environment [3, 4], leading to marginal bone loss [5]. Therefore, the accurate fit between components and the mechanical stability of the prosthetic abutment are paramount for long-term success.

External hexagonal connections present an unfavorable geometry due to the presence of a short support point, which may lead to loosening of the abutment screw when subjected to lateral loads, especially in single-unit restorations [6]. In order to overcome such limitations, alternative connections have been developed, which constitute a stable mechanical system with low-risk of bacterial leakage. Tapered connection implants were therefore introduced as a promising alternative [6].

Several studies evaluating bacterial leakage at the implant/abutment interface of different connection models have shown contamination through the microgap in all implant systems [3, 7–10].

Therefore, the aim of this in vitro study was to compare the effectiveness of the bacterial seal at the implant/abutment interface between two systems, an external hexagon and a tapered implant connection.

## 2. Materials and Methods

Two types of implant-abutment connections manufactured and commercialized in Brazil by the company Intraoss (São Paulo, SP, Brazil) were analyzed. The tapered implant connections were 4.0 mm by 11 mm with a tapered platform of 11.5°, whereas the external hexagons were 3.75 mm by 10 mm (Table 1). The materials used in this study (implants, prosthetic components, and torque wrench) were provided by the manufacturers with no conflict of interests.

**Figure 1:**
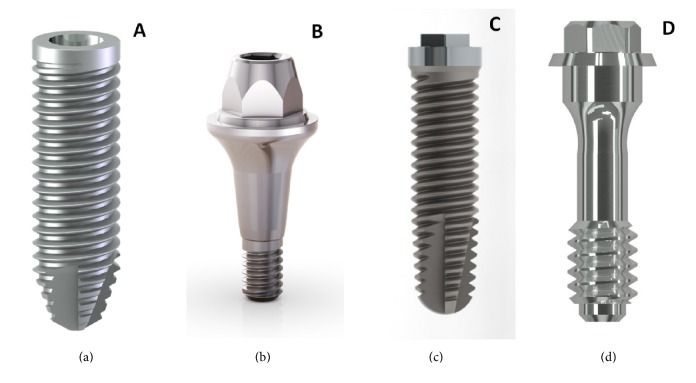
Implants and irrespective prosthetic abutments. Titaoss max CMX (a), universal tapered abutment (b), Titaoss external hexagon (c), and mini abutment EH platform 4.1 (d).

Twelve sets of indexed tapered connection components and twelve sets of external hexagon connection components were used (Figure 1) for microbiological analysis as previously described [10]. For each model, an implant with its respective prosthetic abutment was used as a negative control and a set without the abutment as a positive control of microbial contamination.

The procedures were performed by a single trained and calibrated operator in a laminar flow hood previously sanitized and lined with a sterile sheet. The most apical portion of each of the abutments was contaminated with strains of* Escherichia coli* obtained from ATCC 25922 (American Type Culture Collection, USA). For this purpose, colonies grown on BHI agar (Brain Heart Infusion, Himedia, Mumbai, India) for 24 hours in a bacteriological incubator at 37°C were transferred using rods made from sterilized orthodontic wire with care to avoid contamination of the external surfaces.

The abutment was immediately adapted to the corresponding implant using a manual torque wrench (Intraoss, São Paulo, Brazil) at 32 N·cm following the manufacturer's specifications. The implants were fixed and stabilized with the aid of a sterile bench vise. Each implant-abutment set was introduced into a test tube containing 1 mL of BHI broth into which it was immersed.

In order to ensure a contamination-free outer surface, each specimen was swabbed around the abutment/implant interface using a microbrush dipped in 0.9% saline prior to immersion of the implants in the BHI broth [8]. Each microbrush was also immersed into culture medium as a control for external contamination. As a positive control, an implant from each group was inoculated with* E. coli* strains under the same conditions as described above and immersed in BHI broth without an abutment, following the same culture conditions. As a negative control, an implant from each group was incubated sterile with the connecting prosthetic abutment.

All tubes were identified and kept upright inside a bacteriological incubator for 14 days at 37°C under aerobic conditions. The specimens were checked every 24 hours. Macroscopically, turbidity of the culture broth or deposits at the bottom of the tubes indicated failure of the abutment/implant seal against bacterial leakage. Aliquots were collected from the culture medium inside the test tube (10 *μ*L) from each sample suspicious of being contaminated and seeded onto BHI and incubated in agar at 37°C for 24 hours. This step was used to confirm the findings from the visual macroscopic examination of* E. coli* growth by Gram staining.

After 14 days, the implants were removed from the culture medium and their components were disconnected via countertorque using forceps and the same wrench used for fastening. Failure of the abutment/implant interface seal was observed via turbidity or presence of deposits in the culture.

Descriptive analysis of the data in terms of absolute frequency and relative frequency (percentage) was performed. Fisher's exact test was used at a significance level of 5%.

## 3. Results

Failure of the abutment/implant interface seal was observed via turbidity or presence of deposits in the culture broth in 2/10 (20%) of the external hexagon interface. On the other hand, 0/10 (0%) tapered implant connections were contaminated, though no significant difference was detected between the groups (*p* = 0.4737, Fisher's exact test). No turbidity was observed either in the tubes containing the microbrushes used as the control for abutment/implant interface contamination or in the negative controls.

**Figure 2:**
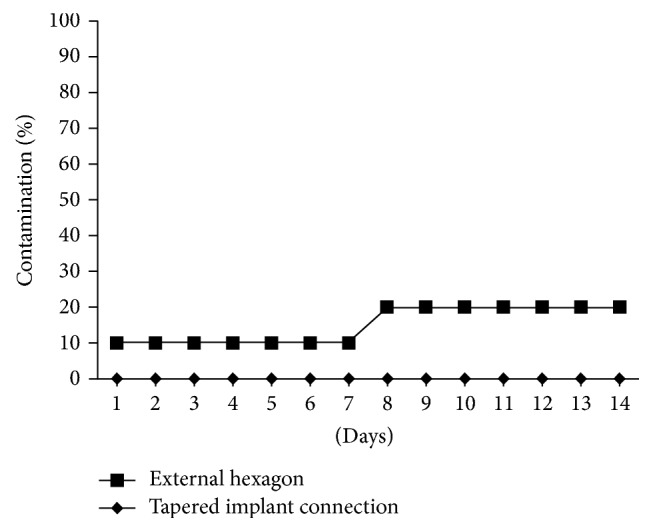
Contamination follow-up expressed as percentage over 14 days.

Figure 2 illustrates the 14-day follow-up of specimen, where the group containing the indexed tapered connection showed no contamination. In the group of external hexagon, one sample was contaminated on the first and the 8th days, whereas two sets (20%) were contaminated at the 14th day.

## 4. Discussion

Alveolar bone loss in peri-implantitis is directly related to bacterial accumulation on the implant surface [11], which is aggravated by the system itself, usually composed of two parts, which favors the appearance of gaps at the implant/abutment interface [1, 2, 12]. Thus, the implant-abutment connection may be considered one of the most important contributing factors to bone loss [13].

A leakage-proof interface with total surface contact between the abutment and the implant has not yet been developed, so that colonization of the internal spaces of the implants by bacteria remains unavoidable [3]. In view of such drawback, the Morse-type connection was introduced, promising a completely stable and movement-free system during the masticatory function, which would result in a reduction of bacterial contamination at this interface [14]. The literature has demonstrated the long-term superiority and predictability of the tapered implant connection when submitted to axial and lateral loads. It has been regarded as a safe, reliable and also an important factor for the maintenance of bone crest, due to microgap reduction and reduced risk of bacterial contamination. Some studies that used different conical fitting systems have, however, demonstrated bacterial leakage through such interface [3, 5, 8, 10, 15].

In view of the diversity of connections available, this in vitro study was carried out to evaluate the microbiological seal of a tapered connection system when compared to the external hexagon system. The findings presented herein demonstrated a low failure rate in the external hexagon system and no failure of the tapered connection system, as far as bacterial leakage was concerned.

Due to the precision of fit at the implant/abutment interface, tapered connections are biomechanically more stable than the external hexagonal [16, 17] preventing the passage and deposition of bacteria [18]. In addition, micromovement of the prosthetic abutment allows undesirable loads to adversely affect esthetics, function and osseointegration [13]. Although the literature highlighted superiority of the tapered implant connection in terms of bacterial sealing, the results obtained in this study demonstrated that both tapered and external hexagonal connections prevented marginal leakage.

It is also important to consider that the use of components from the same manufacturer may improve the stability and sealing of implant systems, which is directly related to the success of implant-based rehabilitations [19]. Additionally, the machining quality minimized micromovements when loading forces are applied.

Therefore, it is presumed that bacterial leakage at the implant-abutment interface occurs as a result of many factors, such as precision of fit and the degree of micromovement between the implant and abutment [20]. In this current study, both implant/abutment connections were able to prevent bacterial leakage in vitro.

## Figures and Tables

**Table 1:** Implants and abutments used.

*Implants*	Batch
External hexagon Titaoss	160100058
Tapered implant connection	150800066

*Abutments*	
Mini abutment HE platform 4.1	150600143
Abutment CMN 2.5 mm	151200026
